# Supplementary material for: Concerted changes in the pediatric single-cell intestinal ecosystem before and after anti-TNF blockade
Source: eLife. 2026 Sep 1;12:RP91792. doi: 10.7554/eLife.91792 (PMC13533482; doi:10.7554/eLife.91792)
Supplement: Supplementary file 2 [file elife-91792-repstand1.docx]

STROBE Statement—checklist of items that should be included in reports of observational studies

|  | Item No. | Recommendation | Page  No. | Relevant text from manuscript |
| --- | --- | --- | --- | --- |
| **Title and abstract** | 1 | (*a*) Indicate the study’s design with a commonly used term in the title or the abstract | 1-2 | treatment-naïve pediatric; diagnostic human biopsies; matched repeat biopsies; non-inflamed pediatric controls |
|  |  | (*b*) Provide in the abstract an informative and balanced summary of what was done and what was found | 1-2 | single-cell transcriptomes; high-resolution scRNA-seq analysis and microscopy; a vector of T cell, innate lymphocyte, myeloid, and epithelial cell states in treatment-naïve pediCD (pediCD-TIME) samples which can distinguish patients along the trajectory of disease severity and anti-TNF response |
| Introduction | | | |  |
| Background/rationale | 2 | Explain the scientific background and rationale for the investigation being reported | 3-6 | **Introduction** |
| Objectives | 3 | State specific objectives, including any prespecified hypotheses | 3-6 | Search for markers of antiTNF treatment refraction |
| Methods | | | |  |
| Study design | 4 | Present key elements of study design early in the paper | 1-6 | FGID controls; repeat biopsies; treatment-naive biopsies stated in abstract and introduction |
| Setting | 5 | Describe the setting, locations, and relevant dates, including periods of recruitment, exposure, follow-up, and data collection | 3-6; 20-25; 29 | Seattle Children’s Hospital; descriptions of timepoints in Intro. Exclusion criteria in Patient cohort details (p29) |
| Participants | 6 | (*a*) *Cohort study*—Give the eligibility criteria, and the sources and methods of selection of participants. Describe methods of follow-up  *Case-control study*—Give the eligibility criteria, and the sources and methods of case ascertainment and control selection. Give the rationale for the choice of cases and controls  *Cross-sectional study*—Give the eligibility criteria, and the sources and methods of selection of participants | 29 | Patient Cohort Details gives eligibility and control selection criteria. More info available on NCT website: <https://clinicaltrials.gov/study/NCT03369353> |
|  |  | (*b*) *Cohort study*—For matched studies, give matching criteria and number of exposed and unexposed  *Case-control study*—For matched studies, give matching criteria and the number of controls per case |  |  |
| Variables | 7 | Clearly define all outcomes, exposures, predictors, potential confounders, and effect modifiers. Give diagnostic criteria, if applicable | 13; 20; 25 | Limitations sections in results and end of discussion (p25) |
| Data sources/ measurement | 8* | For each variable of interest, give sources of data and details of methods of assessment (measurement). Describe comparability of assessment methods if there is more than one group | 28-34 | Methods section details single-cell experimental info, validation experiments, and clinical correlates (microscopy; flow) |
| Bias | 9 | Describe any efforts to address potential sources of bias | 13; 21; 29; 41; 51 | Unsupervised (unbiased) methodologies were adopted for single-cell clustering and other complex analysis; clinical bias control is detailed in patient cohort details |
| Study size | 10 | Explain how the study size was arrived at | 29 | 1 year enrollment and 3 year followup determined cohort sizes |

Continued on next page

| Quantitative variables | 11 | Explain how quantitative variables were handled in the analyses. If applicable, describe which groupings were chosen and why | 13; 21; 29; 41; 51 | Unsupervised (unbiased) methodologies were adopted for single-cell clustering, cluster-free and other complex analysis; we developed ARBOL as an unsupervised clustering method |
| --- | --- | --- | --- | --- |
| Statistical methods | 12 | (*a*) Describe all statistical methods, including those used to control for confounding | 30-59 | Statistical methods are explained in intricate detail in 30 pages of methodology |
|  |  | (*b*) Describe any methods used to examine subgroups and interactions | 30-59 | ARBOL; joint and separate analyses |
|  |  | (*c*) Explain how missing data were addressed | 30-59 | Missing and outlier data are addressed in methods section; especially QC (p32) |
|  |  | (*d*) *Cohort study*—If applicable, explain how loss to follow-up was addressed  *Case-control study*—If applicable, explain how matching of cases and controls was addressed  *Cross-sectional study*—If applicable, describe analytical methods taking account of sampling strategy | 29 | Followup analysis was performed for the subset for which followup was available. |
|  |  | (*e*) Describe any sensitivity analyses | 27; 48; 57 | Sensitivity analysis was performed for predictive models - see pseudo-time bootstrap and RF cross-validation |
| Results | | | | |
| Participants | 13* | (a) Report numbers of individuals at each stage of study—eg numbers potentially eligible, examined for eligibility, confirmed eligible, included in the study, completing follow-up, and analysed | 29 | Patient cohort details |
|  |  | (b) Give reasons for non-participation at each stage | 29 | Patient cohort details |
|  |  | (c) Consider use of a flow diagram | Fig 1a; Table 1 | Figure 1a; Table 1 |
| Descriptive data | 14* | (a) Give characteristics of study participants (eg demographic, clinical, social) and information on exposures and potential confounders | Table 1 | See Table 1 |
|  |  | (b) Indicate number of participants with missing data for each variable of interest | P29 | Patient cohort details |
|  |  | (c) *Cohort study*—Summarise follow-up time (eg, average and total amount) | Table 1; p29 | Patient cohort details; Table 1 |
| Outcome data | 15* | *Cohort study*—Report numbers of outcome events or summary measures over time | 29 | Patient cohort details |
|  |  | *Case-control study—*Report numbers in each exposure category, or summary measures of exposure | *29* | Patient cohort details |
|  |  | *Cross-sectional study—*Report numbers of outcome events or summary measures |  |  |
| Main results | 16 | (*a*) Give unadjusted estimates and, if applicable, confounder-adjusted estimates and their precision (eg, 95% confidence interval). Make clear which confounders were adjusted for and why they were included | S9; S17 | Figure 5-figure supplement 3; Figure 8-figure supplement 2 - bootstraps of pediCD-TIME |
|  |  | (*b*) Report category boundaries when continuous variables were categorized |  |  |
|  |  | (*c*) If relevant, consider translating estimates of relative risk into absolute risk for a meaningful time period |  |  |

Continued on next page

| Other analyses | 17 | Report other analyses done—eg analyses of subgroups and interactions, and sensitivity analyses | 22 | ARBOL + PCA; Fig 5 + 6 |
| --- | --- | --- | --- | --- |
| Discussion | | | | |
| Key results | 18 | Summarise key results with reference to study objectives | 24-25 | pediCD-TIME multiple concerted effects |
| Limitations | 19 | Discuss limitations of the study, taking into account sources of potential bias or imprecision. Discuss both direction and magnitude of any potential bias | 25 | Limitations of the Study section |
| Interpretation | 20 | Give a cautious overall interpretation of results considering objectives, limitations, multiplicity of analyses, results from similar studies, and other relevant evidence | 20-25 | Challenges and Limitations going forward are detailed in discussion |
| Generalisability | 21 | Discuss the generalisability (external validity) of the study results | 25 | Small cohort size limits generalizability - see Limitations section |
| Other information | |  | | |
| Funding | 22 | Give the source of funding and the role of the funders for the present study and, if applicable, for the original study on which the present article is based | 26-27; ICMJE form | Ethics Declaration and ICMJE forms detail funding sources |

*Give information separately for cases and controls in case-control studies and, if applicable, for exposed and unexposed groups in cohort and cross-sectional studies.

**Note:** An Explanation and Elaboration article discusses each checklist item and gives methodological background and published examples of transparent reporting. The STROBE checklist is best used in conjunction with this article (freely available on the Web sites of PLoS Medicine at http://www.plosmedicine.org/, Annals of Internal Medicine at http://www.annals.org/, and Epidemiology at http://www.epidem.com/). Information on the STROBE Initiative is available at www.strobe-statement.org.
