# Supplementary material for: Concerted changes in the pediatric single-cell intestinal ecosystem before and after anti-TNF blockade
Source: eLife. 2026 Sep 1;12:RP91792. doi: 10.7554/eLife.91792 (PMC13533482; doi:10.7554/eLife.91792)
Supplement: Peer review history

# Editor report: eLife Assessment

**DOI:** 10.7554/eLife.91792.3.sa0

**Authors:** Florent Ginhoux

**Affiliations:** Singapore Immunology Network Singapore

**Keywords:** Compelling, Important

This **important** study substantially advances our understanding of pediatric Crohn's disease, mapping the cellular make-up of this disease and how patients respond to treatment. The evidence supporting the conclusions is **compelling**, with thorough bioinformatic analyses, underpinned by rigorous methodology and data integration. The work will be of broad interest to pediatric clinicians, immunologists and bioinformaticians.

# Referee report: Reviewer #1 (Public review)

**DOI:** 10.7554/eLife.91792.3.sa1

Summary:

Crohn's disease is a prevalent inflammatory bowel disease that often results in patient relapse post anti-TNF blockades. This study employs a multifaceted approach utilizing single-cell RNA sequencing, flow cytometry, and histological analyses to elucidate the cellular alterations in pediatric Crohn's disease patients pre and post anti-TNF treatment and comparing them with non-inflamed pediatric controls. Utilizing an innovative clustering approach, , the research distinguishes distinct cellular states that signify the disease's progression and response to treatment. Notably, the study suggests that the anti-TNF treatment pushes pediatric patients towards a cellular state resembling adult patients with persistent relapse. This study's depth offers a nuanced understanding of cell states in CD progression that might forecast the disease trajectory and therapy response.

Robust Data Integration: The authors adeptly integrate diverse data types: scRNA-seq, histological images, flow cytometry, and clinical metadata, providing a holistic view of the disease mechanism and response to treatment.

Novel Clustering Approach: The introduction and utilization of ARBOL, a tiered clustering approach, enhances the granularity and reliability of cell type identification from scRNA-seq data.

Clinical Relevance: By associating scRNA-seq findings with clinical metadata, the study offers potentially significant insights into the trajectory of disease severity and anti-TNF response; might help with the personalized treatment regimens.

Treatment Dynamics: The transition of the pediatric cellular ecosystem towards an adult, more treatment-refractory state upon anti-TNF treatment is a significant finding. It would be beneficial to probe deeper into the temporal dynamics and the mechanisms underlying this transition.

Comparative Analysis with Adult CD: The positioning of on-treatment biopsies between treatment-naïve pediCD and on-treatment adult CD is intriguing. A more in-depth exploration comparing pediatric and adult cellular ecosystems could provide valuable insights into disease evolution.

Areas of improvement:

(1) The legends accompanying the figures are quite concise. It would be beneficial to provide a more detailed description within the legends, incorporating specifics about the experiments conducted and a clearer representation of the data points.

(2) Statistical significance is missing from Fig. 1c WBC count plot, Fig. 2 b-e panels. Please provide even if its not significant. Also, legend should have the details of stat test used.

(3) In the study, the NOA group is characterized by patients who, after thorough clinical evaluations, were deemed to exhibit milder symptoms, negating the need for anti-TNF prescriptions. This mild nature could potentially align the NOA group closer to FIGD-a condition intrinsically defined by its low to non-inflammatory characteristics. Such an alignment sparks curiosity: is there a marked correlation between these two groups? A preliminary observation suggesting such a relationship can be spotted in Figure 6, particularly panels A and B. Given the prevalence of FIGD among the pediatric population, it might be prudent for the authors to delve deeper into this potential overlap, as insights gained from mild-CD cases could provide valuable information for managing FIGD.

(4) Furthermore, Figure 7 employs multi-dimensional immunofluorescence to compare CD, encompassing all its subtypes, with FIGD. If the data permits, subdividing CD into PR, FR, and NOA for this comparison could offer a more nuanced understanding of the disease spectrum. Such a granular perspective is invaluable for clinical assessments. The key question then remains: do the sample categorizations for the immunofluorescence study accommodate this proposed stratification?

(5) The study's most captivating revelation is the proximity of anti-TNF treated pediatric CD (pediCD) biopsies to adult treatment-refractory CD. Such an observation naturally raises the question: How does this alignment compare to a standard adult colon, and what proportion of this similarity is genuinely disease-specific versus reflective of an adult state? To what degree does the similarity highlight disease-specific traits?

Delving deeper, it will be of interest to see whether anti-TNF treatment is nudging the transcriptional state of the cells towards a more mature adult stage or veering them into a treatment-resistant trajectory. If anti-TNF therapy is indeed steering cells toward a more adult-like state, it might signify a natural maturation process; however, if it's directing them toward a treatment-refractory state, the long-term therapeutic strategies for pediatric patients might need reconsideration.

Comments on revisions:

I have no further comments. I am satisfied with the revisions.

# Referee report: Reviewer #2 (Public review)

**DOI:** 10.7554/eLife.91792.3.sa2

Summary:

Through this study the authors combine a number of innovative technologies including scRNAseq to provide insight into Crohn's disease. Importantly, samples from pediatric patients are included. The authors develop a principled and unbiased tiered clustering approach, termed ARBOL. Through high-resolution scRNAseq analysis the authors identify differences in cell subsets and states during pediCD relative to FGID. The authors provide histology data demonstrating T cell localisation within the epithelium. Importantly, the authors find anti-TNF treatment pushes the pediatric cellular ecosystem towards an adult state.

Strengths:

This study is well presented. The introduction clearly explains the important knowledge gaps in the field, the importance of this research, the samples that are used and study design.

The results clearly explain the data, without overstating any findings. The data is well presented. The discussion expands on key findings and any limitations to the study are clearly explained.

I think the biological findings from and bioinformatic approach used in, this study, will be of interest to many and significantly add to the field.

Weaknesses:

(1) The ARBOL approach for iterative tiered clustering on a specific disease condition was demonstrated to work very well on the datasets generated in this study where there were no obvious batch effects across patients. What if strong batch effects are present across donors where PCA fails to mitigate such effects? Are there any batch correction tools implemented in ARBOL for such cases?

The authors have addressed this comment during review

(2) The authors mentioned that the clustering tree from the recursive sub-clustering contained too much noise, and they therefore used another approach to build a hierarchical clustering tree for the bottom-level clusters based on unified gene space. But in general, how consistent are these two trees?

The authors have addressed this comment during review

Comments on revisions:

I have no additional comments. The authors addressed my previous comments well.

# Author comment: Author response

**DOI:** 10.7554/eLife.91792.3.sa3

**Authors:** Hengqi Betty Zheng, Benjamin A Doran, Kyle Kimler, Alison Yu, Victor Tkachev, Veronika Niederlova, Kayla Cribbin, Ryan Fleming, Brandi Bratrude, Kayla Betz, Lorenzo Cagnin, Connor McGuckin, Paula Keskula, Alexandre Albanese, Maria Sacta, Joshua de Sousa Casal, Ruben van Esch, Andrew C Kwong, Conner Kummerlowe, Faith Taliaferro, Nathalie Fiaschi, Baijun Kou, Sandra Coetzee, Sumreen Jalal, Yoko Yabe, Michael Dobosz, Matthew F Wipperman, Sara C Hamon, George D Kalliolias, Andrea Hooper, Wei Keat Lim, Sokol Haxhinasto, Yi Wei, Madeline Ford, Lusine Ambartsumyan, David L Suskind, Dale Lee, Gail H Deutsch, Xuemei Deng, Lauren V Collen, Vanessa Mitsialis, Scott B Snapper, Ghassan Wahbeh, Alex K Shalek, José Ordovas-Montanes, Leslie S Kean

**Affiliations:** Seattle Children's Hospital Seattle United States; Massachusetts Institute of Technology Boston United States; Boston Children's Hospital Boston United States; Boston Children's Hospital Boston United States; Boston Children's Hospital Boston United States; Boston Children's Hospital Boston United States; Seattle Children's Hospital Seattle United States; Boston Children's Hospital Boston United States; Boston Children's Hospital Boston United States; Boston Children's Hospital Boston United States; Boston Children's Hospital Boston United States; Boston Children's Hospital Boston United States; Boston Children's Hospital Boston United States; Boston Children's Hospital Boston United States; Boston Children's Hospital Boston United States; Boston Children's Hospital Boston United States; Boston Children's Hospital Boston United States; Boston Children's Hospital Boston United States; Massachusetts Institute of Technology Cambridge United States; Boston Children's Hospital Boston United States; Regeneron Tarrytown United States; Regeneron Tarrytown United States; Regeneron Tarrytown United States; Regeneron Tarrytown United States; Regeneron Tarrytown United States; Regeneron Tarrytown United States; Regeneron Tarrytown United States; Regeneron Tarrytown United States; Regeneron Tarrytown United States; Regeneron Andrea Hooper United States; Regeneron Tarrytown United States; Regeneron Tarrytown United States; Regeneron Tarrytown United States; Seattle Children's Hospital Seattle United States; Seattle Children's Hospital Seattle United States; Seattle Children's Hospital Seattle United States; Seattle Children's Hospital Seattle United States; Seattle Children's Hospital and University of Washington Seattle United States; Seattle Children's Hospital Seattle United States; Boston Children's Hospital Boston United States; Brigham and Women's Hospital Boston United States; Boston Children's Hospital Boston United States; Seattle Children's Hospital Seattle United States; Massachusetts Institute of Technology Cambridge United States; Boston Children's Hospital Boston United States; Boston Children's Hospital Boston United States

The following is the authors’ response to the original reviews.

**Reviewer #1 (Public Review):**

Summary:

Crohn's disease is a prevalent inflammatory bowel disease that often results in patient relapse post anti-TNF blockades. This study employs a multifaceted approach utilizing single-cell RNA sequencing, flow cytometry, and histological analyses to elucidate the cellular alterations in pediatric Crohn's disease patients pre and post-anti-TNF treatment and comparing them with non-inflamed pediatric controls. Utilizing an innovative clustering approach, the research distinguishes distinct cellular states that signify the disease's progression and response to treatment. Notably, the study suggests that the anti-TNF treatment pushes pediatric patients towards a cellular state resembling adult patients with persistent relapses. This study's depth offers a nuanced understanding of cell states in CD progression that might forecast the disease trajectory and therapy response.

Robust Data Integration: The authors adeptly integrate diverse data types: scRNA-seq, histological images, flow cytometry, and clinical metadata, providing a holistic view of the disease mechanism and response to treatment.

Novel Clustering Approach: The introduction and utilization of ARBOL, a tiered clustering approach, enhances the granularity and reliability of cell type identification from scRNA-seq data.

Clinical Relevance: By associating scRNA-seq findings with clinical metadata, the study offers potentially significant insights into the trajectory of disease severity and anti-TNF response; which might help with the personalized treatment regimens.

Treatment Dynamics: The transition of the pediatric cellular ecosystem towards an adult, more treatment-refractory state upon anti-TNF treatment is a significant finding. It would be beneficial to probe deeper into the temporal dynamics and the mechanisms underlying this transition.

Comparative Analysis with Adult CD: The positioning of on-treatment biopsies between treatment-naïve pediCD and on-treatment adult CD is intriguing. A more in-depth exploration comparing pediatric and adult cellular ecosystems could provide valuable insights into disease evolution.

Areas of improvement:

(1) The legends accompanying the figures are quite concise. It would be beneficial to provide a more detailed description within the legends, incorporating specifics about the experiments conducted and a clearer representation of the data points.

We agree that it is beneficial to have descriptive figure legends that balance elements of experimental design, methodology, and statistical analyses employed in order to have a clear understanding throughout the manuscript. We have gone through and clarified areas throughout.

(2) Statistical significance is missing from Fig. 1c WBC count plot, Fig. 2 b-e panels. Please provide it even if it's not significant. Also, the legend should have the details of stat test used.

We have now added details of statistical significance data in the Figure 1 legends. Please note that Mann-Whitney U-test was used for clinical categorical data.

(3) In the study, the NOA group is characterized by patients who, after thorough clinical evaluations, were deemed to exhibit milder symptoms, negating the need for anti-TNF prescriptions. This mild nature could potentially align the NOA group closer to FGID-a condition intrinsically defined by its low to non-inflammatory characteristics. Such an alignment sparks curiosity: is there a marked correlation between these two groups? A preliminary observation suggesting such a relationship can be spotted in Figure 6, particularly panels A and B. Given the prevalence of FGID among the pediatric population, it might be prudent for the authors to delve deeper into this potential overlap, as insights gained from mild-CD cases could provide valuable information for managing FGID.

Thank you for this insightful point. On histopathology and endoscopy, the NOA exhibited microscopic and macroscopic inflammation which landed these patients with the CD diagnosis, albeit mild on both micro and macro accounts. By contrast, the FGID group by definition will not have inflammation of microscopic and macroscopic evaluation. There is great interest in the field of adult and pediatric gastroenterology to understand why patients develop symptoms without evidence of inflammation. However, in 2023 the diagnostic tools of endoscopy with biopsy and histopathology is not sensitive enough to detect transcript level inflammation, positioning single-cell technology to be able to reveal further information in both disease processes.

Based on the reviewer’s suggestions, we have calculated a heatmap of overlapping NOA and FGID cell states along the Figure 6a joint-PC1, showing where NOA CD patients and FGID patients overlap in terms of cell states. This is displayed in Supplemental Figure 15d. This revealed a set of T, Myeloid, and Epithelial cell states that were most important in describing variance along the FGID-CD axis, allowing us to hone in on similarities at the boundary between FGID and CD. By comparing the joint cell states with CD atlas curated cluster names, we identified CCR7-expressing T cell states and GSTA2-expressing epithelial states associated with this overlap.

(4) Furthermore, Figure 7 employs multi-dimensional immunofluorescence to compare CD, encompassing all its subtypes, with FGID. If the data permits, subdividing CD into PR, FR, and NOA for this comparison could offer a more nuanced understanding of the disease spectrum. Such a granular perspective is invaluable for clinical assessments. The key question then remains: do the sample categorizations for the immunofluorescence study accommodate this proposed stratification?

Thank you for the thoughtful discussion. We agree that stratifying Crohn’s disease by PR, FR, and NOA would provide valuable clinical insight. Unfortunately our multiplex IF cohort was designed to maximize overall CD versus FGID comparisons and does not contain enough samples in patient subgroups to power such an analysis. We have highlighted this limitation in the text.

(5)The study's most captivating revelation is the proximity of anti-TNF-treated pediatric CD (pediCD) biopsies to adult treatment-refractory CD. Such an observation naturally raises the question: How does this alignment compare to a standard adult colon, and what proportion of this similarity is genuinely disease-specific versus reflective of an adult state? To what degree does the similarity highlight disease-specific traits?

Delving deeper, it will be of interest to see whether anti-TNF treatment is nudging the transcriptional state of the cells towards a more mature adult stage or veering them into a treatment-resistant trajectory. If anti-TNF therapy is indeed steering cells toward a more adult-like state, it might signify a natural maturation process; however, if it's directing them toward a treatment-refractory state, the long-term therapeutic strategies for pediatric patients might need reconsideration.

Thank you to the reviewer for another insightful point. We agree that age-matched samples are critical to evaluate disease cell states and hence we have age-matched controls in our pediatric cohort. Our timeline of follow-up only spans 3 years and patients remain in the pediatric age range at times of follow-up endoscopy and biopsy and would not be reflective of an adult GI state. We believe that the cellular behavior from naïve to treatment biopsy to on treatment biopsy is reflective of disease state rather than movement towards and adult-like state. We would also like to point out that pediatric onset IBD (Crohn’s and ulcerative colitis) traditionally has been harder to treat and presents with more extensive disease state (PMID: 22643596) and the ability to detect need for therapy escalation/change would be an invaluable tool for clinicians.

We share the reviewer’s interest in disentangling a natural maturation process from disease and treatment-specific changes. Because the patients who were not given treatment did not move towards the adult-like phenotype, it could point to a push towards a treatment-resistant trajectory. To further support these findings, we generated a new disease-pseudotime figure Supplemental Figure 17, using cross-validation methods and the TradeSeq package. This figure was designed to track how each pediatric sample shifts from the treatment-naïve state through antiTNF therapy and to test the robustness of these shifts across samples. The new visualizations show patterns that do not recapitulate natural aging processes but rather shifts across all cell types associated with antiTNF treatment.

**Reviewer #2 (Public Review):**

Summary:

Through this study, the authors combine a number of innovative technologies including scRNAseq to provide insight into Crohn's disease. Importantly samples from pediatric patients are included. The authors develop a principled and unbiased tiered clustering approach, termed ARBOL. Through high-resolution scRNAseq analysis the authors identify differences in cell subsets and states during pediCD relative to FGID. The authors provide histology data demonstrating T cell localisation within the epithelium. Importantly, the authors find anti-TNF treatment pushes the pediatric cellular ecosystem toward an adult state.

Strengths:

This study is well presented. The introduction clearly explains the important knowledge gaps in the field, the importance of this research, the samples that are used, and study design.

The results clearly explain the data, without overstating any findings. The data is well presented. The discussion expands on key findings and any limitations to the study are clearly explained.

I think the biological findings from, and bioinformatic approach used in this study, will be of interest to many and significantly add to the field.

Weaknesses:

(1) The ARBOL approach for iterative tiered clustering on a specific disease condition was demonstrated to work very well on the datasets generated in this study where there were no obvious batch effects across patients. What if strong batch effects are present across donors where PCA fails to mitigate such effects? Are there any batch correction tools implemented in ARBOL for such cases?

We thank the reviewer for their insightful point, the full extent to which ARBOL can address batch effects requires further study. To this end we integrated Harmony into the ARBOL architecture and used it in the paper to integrate a previous study with the data presented (Figure 8). We have added to ARBOL’s github README how to use Harmony with the automated clustering method. With ARBOL, as well as traditional clustering methods, batch effects can cause artifactual clustering at any tier of clustering. Due to iteration, this can cause batch effects to present themselves in a single round of clustering, followed by further rounds of clustering that appear highly similar within each batch subset. Harmony addresses this issue, removing these batch-related clustering rounds. The later arrangement of fine-grained clusters using the bottom-up approach can use the batch-corrected latent space to calculate relationships between cell states, removing the effects from both sides of the algorithm. As stated, the extent to which ARBOL can be used to systematically address these batch effects requires further research, but the algorithmic architecture of ARBOL is well suited to address these effects.

(2) The authors mentioned that the clustering tree from the recursive sub-clustering contained too much noise, and they therefore used another approach to build a hierarchical clustering tree for the bottom-level clusters based on unified gene space. But in general, how consistent are these two trees?

Thank you for this thoughtful question. The two tree methodologies are not consistent due to their algorithmic differences, but both are important for several reasons:

(1) The clustering tree is top-down, meaning low resolution lineage-related clusters are calculated first. Doublets and quality differences can cause very small clusters of different lineages (endothelial vs fibroblast) to fall under the incorrect lineage at first in the sub clustering tree, but these are recaptured during further sub clustering rounds, and then disentangled by the cluster-centroid tree.

(2) The hierarchical tree is a rose tree, meaning each branching point can contain several daughter branches, while taxonomies based on distances between species (or cell types in this case) are binary trees with only 2 branches per branching point, because distances between each cluster are unique. Because this taxonomy, or bottom-up, is different from the top-down approach, it is useful to then look at how these bottom-level clusters are similar. To that end, we performed pair-wise differential expression between all end clusters and clustered based on those genes.

(3) Calculation of a binary tree represents a quantitative basis for comparing the transcriptomic distance between clusters as opposed to relying on distances calculated within a heuristic manifold such as UMAP or algorithmic similarity space such as cluster definitions based on KNN graphs.

In practice, this dual view rescues small clusters that may have been mis-grouped by technical artifacts and gives a quantitative distance based hierarchy that can be compared across metadata covariates.
